# Alkaloids from Marine Ascidians

**DOI:** 10.3390/molecules16108694

**Published:** 2011-10-19

**Authors:** Marialuisa Menna, Ernesto Fattorusso, Concetta Imperatore

**Affiliations:** The NeaNat Group, Dipartimento di Chimica delle Sostanze Naturali, Università degli Studi di Napoli “Federico II”, Via D. Montesano 49, 80131, Napoli, Italy

**Keywords:** natural products, alkaloids, ascidians, ecteinascidis, lamellarins

## Abstract

About 300 alkaloid structures isolated from marine ascidians are discussed in term of their occurrence, structural type and reported pharmacological activity. Some major groups (e.g., the lamellarins and the ecteinascidins) are discussed in detail, highlighting their potential as therapeutic agents for the treatment of cancer or viral infections.

## 1. Introduction

Ascidians belong to the phylum Chordata, which encompasses all vertebrate animals, including mammals and, therefore, they represent the most highly evolved group of animals commonly investigated by marine natural products chemists. Together with the other classes (Thaliacea, Appendicularia, and Sorberacea) included in the subphylum Urochordata (=Tunicata), members of the class Ascidiacea are commonly referred to as tunicates, because their body is covered by a saclike case or tunic, or as sea squirts, because many species expel streams of water through a siphon when disturbed. There are roughly 3,000 living species of tunicates, of which ascidians are the most abundant and, thus, the mostly chemically investigated. An interest in ascidians’ chemistry was kindled as early as 1847, when a German physiologist discovered the presence in the blood cells of these invertebrates of large amounts of vanadium and sulfuric acid, along with an uncharacterized nitrogenous metabolite. The conquest to understand the role of vanadium in ascidians’ blood has led to a huge amount of chemical and biological studies and led to the isolation very unstable hydroquinoid compounds, called tunichromes [1,2], whose biological function as well as functional role in the vanadium complexation remains still unclear [3,4]. Then, attention has focused on ascidians because of their biologically active metabolites and the chemistry of ascidians has become one of the most active fields of marine natural products; it has been amply demonstrated that these sea creatures are prolific producers of unusual structures with significant bioactivities [5,6].

Ascidians’ chemistry is dominated by the presence of nitrogenous metabolites which could be basically divided into two structural type-based groups, peptides and polycyclic aromatic alkaloids. This review describes the chemistry of ascidians’ alkaloids; its overall goal is to display the huge chemical diversity in this group of marine secondary metabolites. Ascidians’ alkaloids class indeed includes a large variety of structures, ranging from complex pyridoacridines and tyrosine-derived alkaloids to simple protoalkaloids. About 300 alkaloid structures isolated from marine ascidians will be discussed in term of their occurrence, structural type and reported pharmacological activity. They will be presented in an order based on their general structural type or on a plausible biosynthetic origin (amino acid origin). Some major groups (e.g., the lamellarins and the ecteinascidins) will be discussed in detail, highlighting their potential as therapeutic agents for the treatment of cancer or viral infections.

## 2. Pyridoacridine Alkaloids

Pyridoacridine alkaloids isolated from ascidians are typically *tetra*- or *penta*-cyclic aromatic alkaloids based on the pyrido[*k,l*]acridine skeleton, usually possessing a functionalized alkylamine side chain. Many of these compounds have generated interest both as challenging problems for structure elucidation and synthesis as well as for their bioactivities. In general, pyridoacridines are cytotoxic and some of them possess potent anti-viral, anti-fungal, anti-bacterial, anti-tumor and anti-parasitic activity [7]. For the majority of this class, cytotoxicity has been shown to be due to DNA-binding properties, topoisomerase (TOPO) inhibition or the production of reactive oxygen species (ROS) [7]. In fact, the main structural feature of these alkaloids is the core of a planar iminoquinone moiety which can intercalate into DNA and cleave the DNA double helix or inhibit the action of TOPO II. Thus, there has been considerable interest on the potential of these compounds as antitumor agents [8,9]; the great chemical diversity in this family of alkaloids provided a large set of tools to manipulate the TOPO II activity, either stimulating cleavage of DNA through stabilization of covalent TOPO II-DNA complexes or promoting the catenation of DNA [10]. During the past 25 years, both natural pyridoacridines and their analogues have constituted excellent targets for synthetic works [9], confirming that this family of alkaloids as a whole is of interest as a source of new lead structures for drug development.

The known tetracyclic pyridoacridine alkaloids from marine sources are dominated by those isolated from ascidians. They show an oxygen function at C-8, which can be a carbonyl group, a hydroxyl, or an ether moiety. Pantherinine (**1**), isolated from *Aplidium pantherinum * [11] and cystodytins A–K (**2–12**), from an Okinawan *Cystodytes dellechiajei * [12,13,14,15], belong to the iminoquinone series. Cystodytins were shown to possess, in addition to a potent cytotoxic activity, powerful Ca^2+^-releasing activity in the sarcoplasmatic reticulum [12]. Diplamines A (**13**), from a *Diplosoma* sp. [16], and B (**14**), from *Lissoclinum *cf. *badium * [17], possess a thiomethyl functionality on the aromatic nucleus at C-9, which seems critical for cytotoxicity; furthermore, movement of this function from C-9 to C-5 in isodiplamine (**15**), from *L. notti * [15], reduces cytotoxicity. Strictly related to diplamines are lissoclins A and B (**16** and **17**), from an Australian *Lissoclinum *sp. [18]; unfortunately, the cytotoxic properties of these two alkaloids have not been investigated.

**Figure 1:**
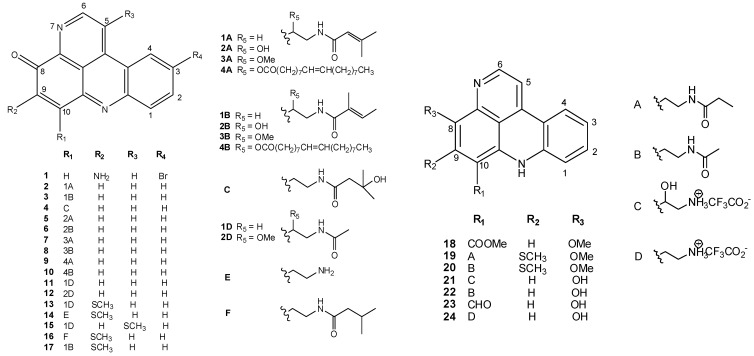
Tetracyclic pyridoacridine alkaloids from ascidians.

Nor-segoline (**18**), from a purple tunicate from the Red Sea, *Eudistoma *sp. [19,20], varamines **19** and **20**, from a Fijian collection of *L. vareau * [21], and styelsamines **21–24**, from an Indonesian collection of *Eusynstela latericius * [22], possess the 11*H*-pyrido[4,3,2-*m,n*]acridine nucleus; nor-segoline exhibited potent antileukemic properties [23] and varamines were strongly cytotoxic (Figure 1).

**Figure 2:**
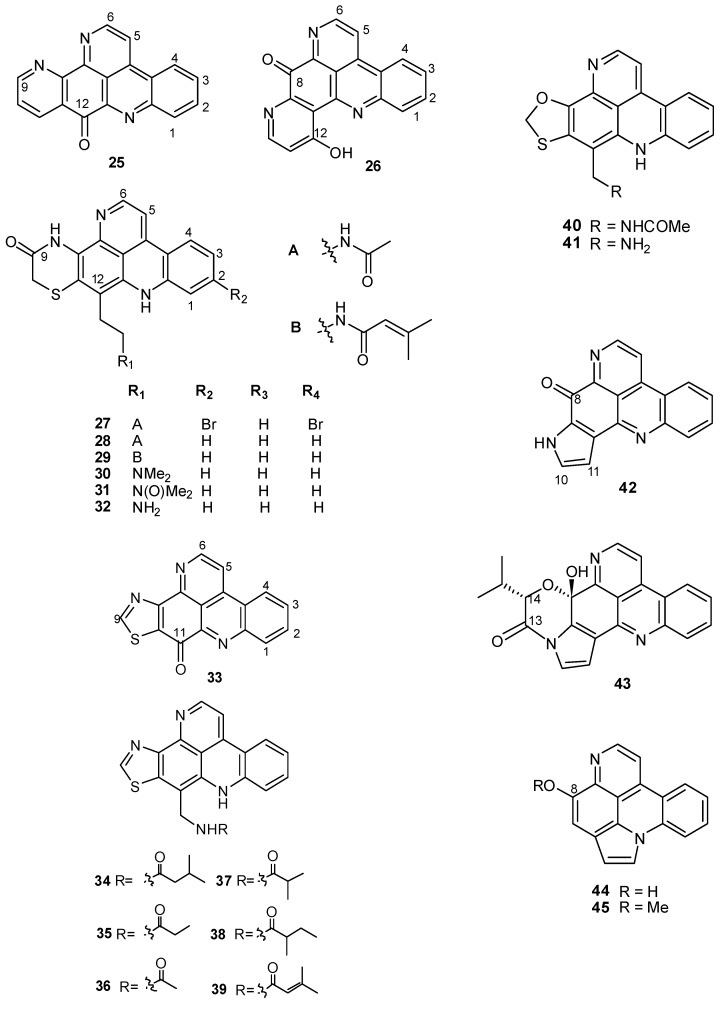
Pentacyclic pyridoacridine alkaloids from ascidians.

Pentacyclic pyridoacridines show an additional heterocyclic ring, generally fused at C9,10 or at C8,9 of the acridine ring C. For example, ascididemin (**25**), from a *Didemnum* sp. [24], and meridine (**26**) [25], from *Amphicarpa meridiana*, highly cytotoxic alkaloids, both contain a further pyridine moiety. The shermilamines **27–32**, isolated from a *Trididemnum* sp., are thiazinone-containing pentacyclic alkaloids differing in their C-12 side chains [26,27,28,29]. A series of thiazole-containing compounds, kuanoniamines A–F (**33–39**), have been isolated from both an unidentified Micronesian ascidian and the Lamellariidae mollusk *Chelynotus semperi*, believed to be a predator of the ascidian [30,31]. Kuanoniamines B–F (**34–39**) differ in their acylated phenethylamine side chains, while structure of kuanoniamine A possesses the same tetracyclic iminoquinone-containing pyridoacridine ring system found in ascididemin (**25**). Lissoclinidines **40**, **41**, isolated from ascidians of *Lissoclinum *genus [15,17], feature a benzoxathiole moiety, while both sebastianines **42** and **43**, from a Brazilian collection of *Cystodites dellechiaiei * [32], and arnoamines **44** and **45**, from a *Cystodites *sp. [33], contain a pyrrole ring (Figure 2).

**Figure 3:**
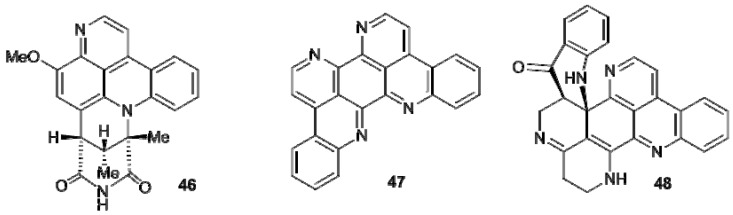
Structures of segoline A (**46**), eilatin (**47**), and eudistone A (**48**).

Segolines (**46**, segoline A), possessing a benzo-1,6-diazaphenantroline ring system, eilatin (**47**), having an unusual symmetrical heptacyclic structure, and eudistones (**48**, eudistone A) are more complex pyridoacridines (Figure 3). All these alkaloids have been isolated from *Eudistoma* ascidians [18,19,34,35,36] and have been shown to be potent regulators of cellular growth and differentiation and affect cAMP-mediated processes [37]. Eilatin (**47**) can form complexes with metal ions and these complexes represent a new family of compounds with unusual nucleic acid binding specificity [38] (Figure 3).

## 3. Carboline-Based Alkaloids

Polysubstituted β-carbolines, as well as dihydro-, and tetrahydro-β-carbolines form a large group of tryptophan-derived ascidian metabolites. The majority of these alkaloids have been isolated from tunicates belonging to the genus *Eudistoma* [39,40,41,42,43,44,45,46,47,48,49,50,51,52], but other sources are the genera *Ritterella * [53,54], * Pseudodistoma * [55,56,57], * Didemnum * [58,59,60,61,62], *Synoicum * [63], and *Lissoclinum * [64,18]. Almost all the reported compounds are hydroxylated and/or brominated at C5, C6, C7, and C8; apart from few members of the group, which are unsubstituted at C1, they show different substituents at this position, such as pyrrole, pyrroline, or indole rings, as well as oxygenated, aminatated, or thiomethylated alkyl residues.

Actually, all of the β-carboline metabolites isolated thus far are related biosynthetically; the production of these metabolites is generally believed to involve the coupling of tryptophan with a second amino acid, whose nature clearly affects C1 substitution. For example, eudistomins A (**54**), M (**55**), G (**50**), and P (**52**) may be considered to be derived from tryptophan and glutamine [39], while the 2-fenylacetyl-β-carbolines, eudistomines R (**56**) and T (**57**), are, in addition to tryptophan, made up of phenylalanine or phenylpyruvic acid. Similarly, the unusual aminoacids, *p*-methylphenyl-L-alanine and *S*-methyl-D-cysteine have been supposed to be involved in the production of eudistomidins B (**83**) and C (**64**) [46], respectively. A proline-derived precursor is evident in eudistomins G, I, P (**50–52**), A (**54**), and M (**55**), eudistomidin A (**63**), and woodinine (**85**). *In vivo* studies with *E. olivaceum* confirmed that tryptophan and proline are the primary precursors of eudistomin I (**51**) [65]. Some of the most intriguing members of these alkaloids are those bearing an oxathiazepine ring (eudistomins C, E, F, K, and L, **91–95**) which apparently originate from condensation of tryptophan with cysteine [44]. Eudistalbins A (**65**) and B (**66**) likely derive from leucine.

**Figure 4:**
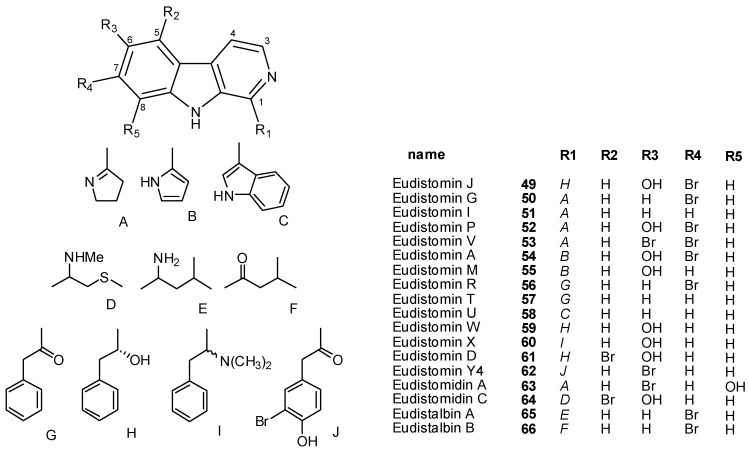
Simple β-carbolines.

Examples of simple β-carboline alkaloids are the eudistomins **49–62** [39,40,41,42,43], eudistomidins A (**63**) and C (**64**) [45,46], and eudistalbins **65** and **66** [49]. Eudistomidin A is the first calmodulin antagonist from marine origin (Figure 4) [45].

**Figure 5:**
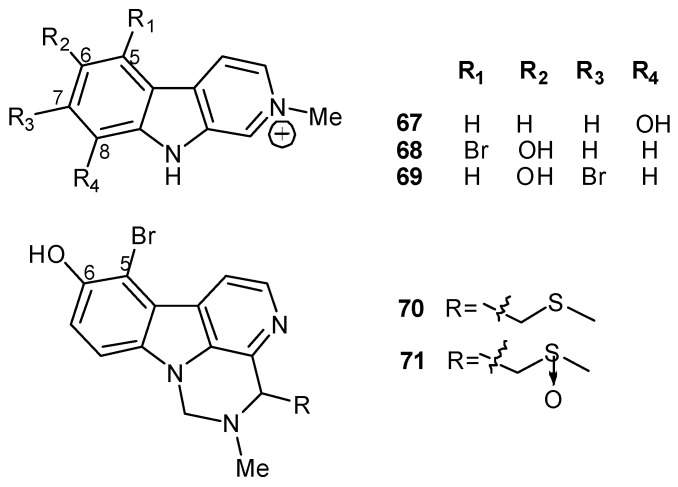
*N*-methyl β-carbolinium (**67–69**) and tetrahydropyrimidine ring containing (**70** and **71**) β-carboline derivatives.

Pseudodistamine (**67**) [57] as well as 2-methyleudistomins D (**68**) and J (**69**) [50] are rare examples of *N*-methyl β-carbolinium derivatives described from marine sources, while eudistomidins E (**70**) and F (**71**) are structurally unique, with a tetrahydropyrimidine ring fused to the β-carboline skeleton (Figure 5) [47].

**Figure 6:**
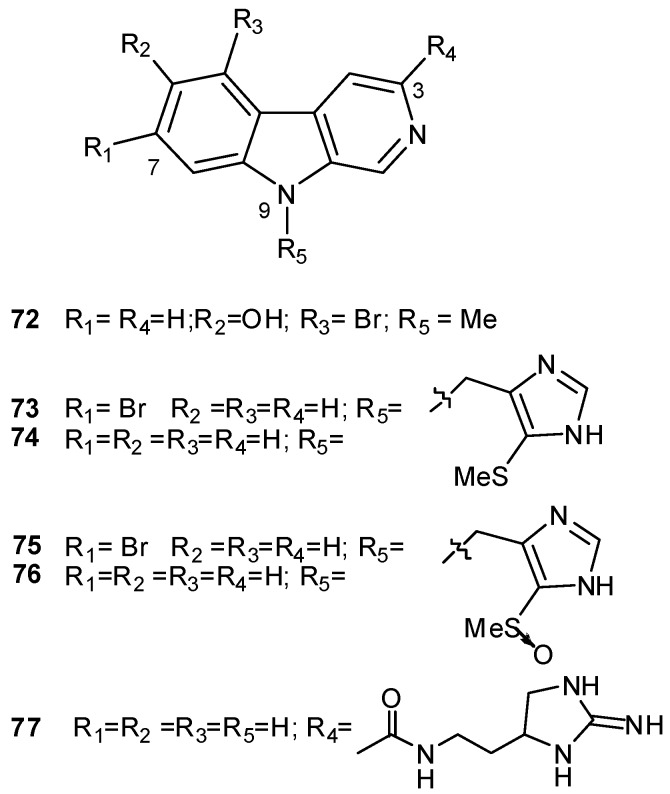
N9-(**72–76**) and C3-substituted (**77**) β-carbolines.

**Figure 7:**
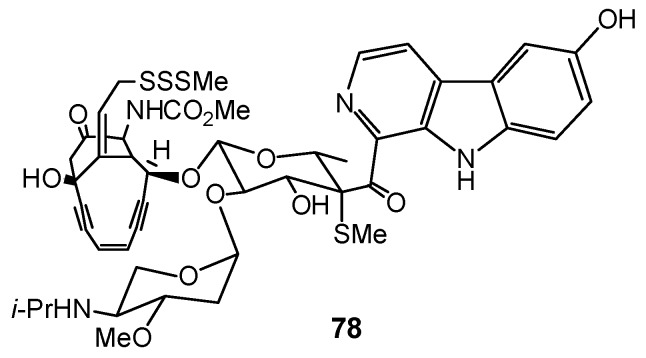
Structures of shishijimicin A (**78**).

Eudistomidin D (**72**) as well as didemnolines A–D **73–76** differ from the other ascidian β-carboline metabolites in that they are substituted at the N9 position of the β-carboline ring, rather than at the C1 position [46,58]. Tiruchanduramide (**77**) is a β-carboline guanidine alkaloid and it is the sole 3-substituted β-carboline isolated to date from ascidians (Figure 6) [63]. Shishijimicins (shishijimicin A, **78**) are perhaps the most complex β-carbolines from ascidians; they belong to the enediyne class of antibiotics, which are also potent antitumor agents (Figure 7) [59].

**Figure 8:**
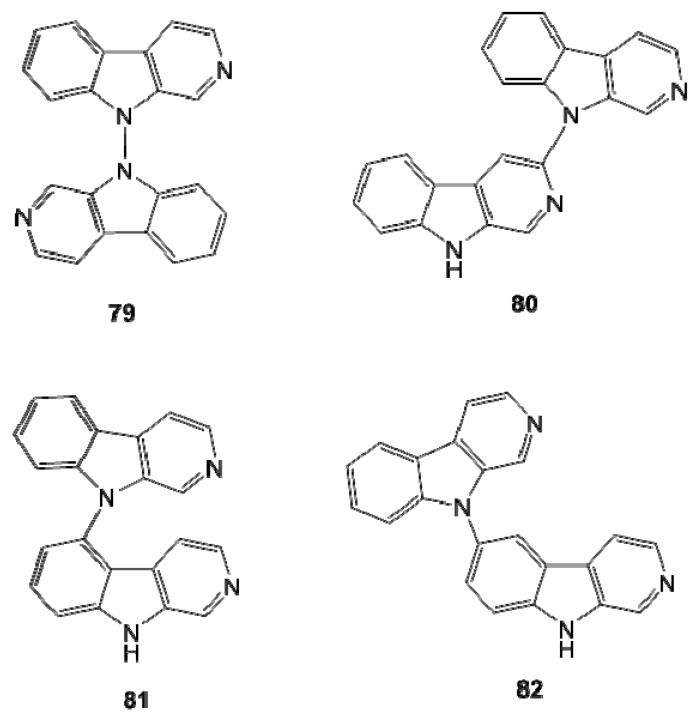
Symmetrical (**79**) and non-symmetrical (**80–82**) β-carboline dimers.

Both symmetrical (**79**) and non-symmetrical β-carboline dimers (**80–82**) have been reported from *Didemnum* sp. and their structures were confirmed by synthesis [60,61]. Compound **79** was known as a synthetic compound derived from photochemical dimerization of β-carboline (norharmane) but it is the first example of naturally occurring β-carboline dimer (Figure 8).

**Figure 9:**
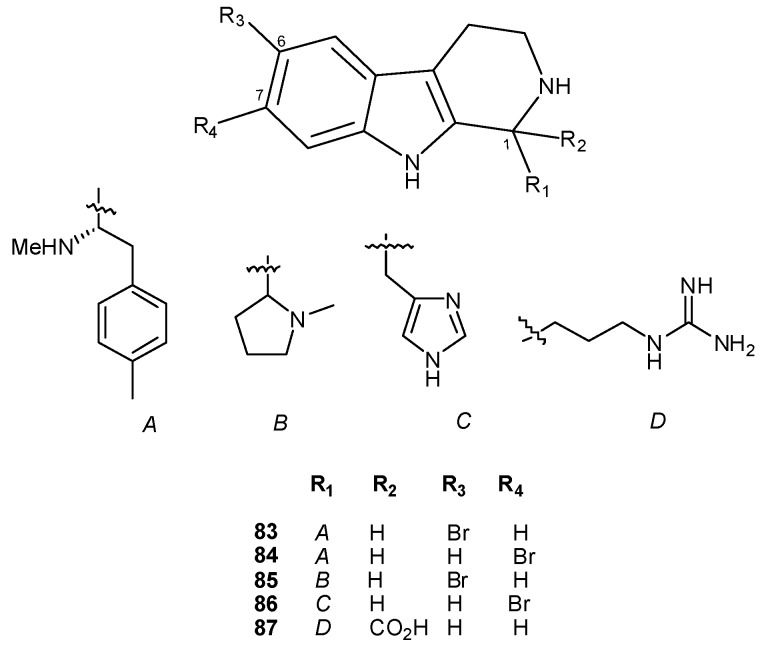
Tetrahydro-β-carbolines.

Numerous tetrahydro-β-carbolines with various substituents at C1 have been reported from ascidians; they include eudistomidins B (**83**) and G (**84**) [46,48], woodinine (**85**) [51], lissoclin C (**86**) [18], and compound **87**, the 1-carboxyl analogue of trypargine, a tetrahydro-β-carboline previously isolated from the skin of the African frog *Kassina senegalensi *(Figure 9) [52].

**Figure 10:**
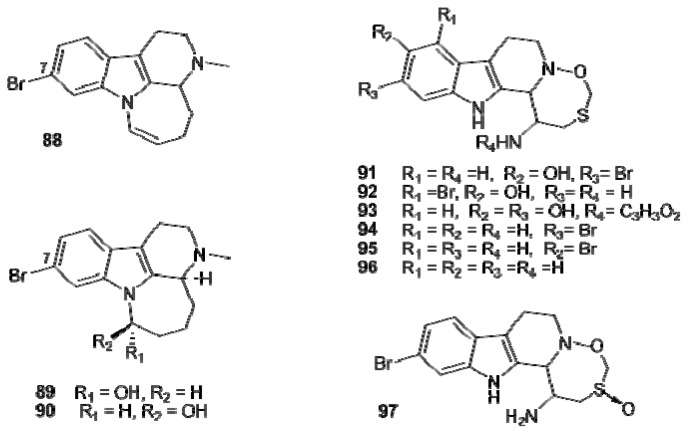
7-Membered additional ring containing tetrahydro β-carbolines.

**Figure 11:**
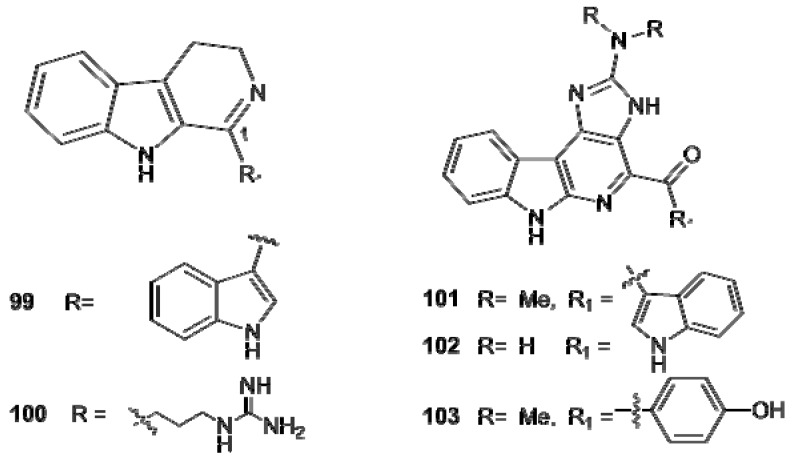
Structure of bengacarboline (**98**).

Arborescidines B–D (**88–90**) [56], eudistomins C, E, F, K, L (**91–95**) [44], K-sulfoxide (**97**) [53], and debromoeudistomin K (**96**) [54] contain a 7-membered additional ring attached to the tetrahydro β-carboline moiety which, in the case of the eudistomins, is an oxathiazepine ring (Figure 10). Bengacarboline (**98**) is a tetrahydro-β-carboline with two indole units attached to C1 of the carboline nucleus (Figure 11); it resulted cytotoxic toward a 26 cell line human tumor panel *in vitro* and inhibited the catalytic activity of TOPO II [62].

**Figure 12:**
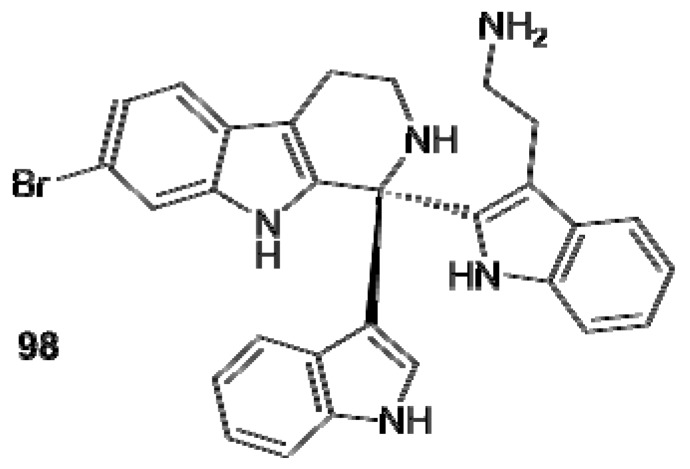
Dihydro-β-carbolines (**99**, **100**) and α-carbolines (**101–103**).

The β-carboline alkaloids display a variety of biological activities, including a broad spectrum of antibiotic activity, cytotoxicity, calmodulin antagonistic properties. However, the most notable pharmacological feature is the strong antiviral properties displayed by some members of the family [66,67]. The tetrahydro-β-carbolines generally exhibited higher levels of biological activity than their fully aromatic relatives; the oxathiazepino-eudistomins **88–94**, for example, exhibit the highest level of antiviral activity and were also endowed with antimicrobial activity. Eudistomin K (**91**) is significantly active against *Herpes simplex* Type I (HSV-1) and *Polio* virus, while the sulfoxide (**94**) and debromo (**90**) derivatives are active against both virus but less potent. Enhancement of antiviral activity correlates to bromination at C7 (Figure 12).

There are only few examples of reported dihydro-β-carbolines from ascidians. The first was isoeudistomin U (**99**) which was initially reported to be a 4-substituted dihydro-α-carboline derivative [64], but whose structure was revised to 3,4-dihydroeudistomin U after total synthesis [68]; the bioactivity of isoeudistomin U was reported to be similar to that of the related eudistomin U (**59**). A 1,2-dehydro analogue of trypargine, named trypargimine (**100**), has been reported from a previously undescribed *Eudistoma* sp. [52]. Also α-carbolines are not common ascidians’ metabolites. The grossularines (**101–103**) from *Dendrodoa grossularia * are the sole representatives of this structural class and are the first α-carbolines to be isolated from a natural source (Figure 12). The initial structure reported for grossularine (=grossularine-1) [69] was incorrect and it was revised when data for both grossularine-1 (**101**) and -2 (**103**) were reported [70]. Both grossularines are cytotoxic and cause accumulation of cells in the G1-phase; grossularine-2 appears to act on DNA as a mono-intercalating agent. *N,N*-didesmethylgrossularine-1 (**102**) has been reported from *Polycarpa aurata* but no activity data were presented [71].

## 4. Indole-Based Alkaloids

**Figure 13:**
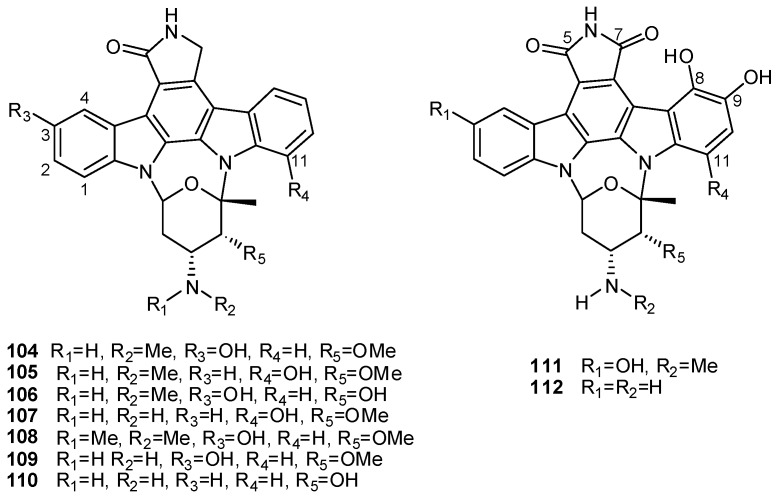
Staurosporine derivatives **104–112**.

The structures of indole based alkaloids isolated from ascidians span a wide range of complexity, spreading from the simple 6-bromoindole-3-carbaldehyde, isolated from *Pyura sacciformis * [72] and previously found in a marine pseudomonad [73], to the complex indolocarbazoles of the staurosporine type **104–112** isolated from ascidians belonging to the family Polycitoridae (Figure 13) [74,75,76,77,78,79]*.* Staurosporine and its derivatives have been isolated from various actinomycete strains as well as from some marine organisms, including ascidians [80]. These alkaloids were shown to be strong inhibitors of several kinases, in particular protein kinase C (PKC); other activities include inhibition of platelet aggregation and smooth muscle contraction, induction of cell cycle arrest and apoptosis, and the reversal of multidrug resistance in some cancer cell lines. The potential of these derivatives as anticancer agents is supported by the example of 7-hydroxystaurosporine which is in clinical phase I trials at the NCI [77]. 11-Hydroxystaurosporine (**105**), from *Eudistoma* sp., is a PKC inhibitor about 30% more potent than staurosporine [74] whereas 3-hydroxystaurosporine (**104**), from *E. toealensis*, is one of the most active staurosporine-type inhibitors of cell proliferation described so far [77]. Derivatives **111** and **112**, from *Cystodites solitus*, displayed cytotoxic activity in the submicromolar range against three human tumour cell lines [79].

**Figure 14:**
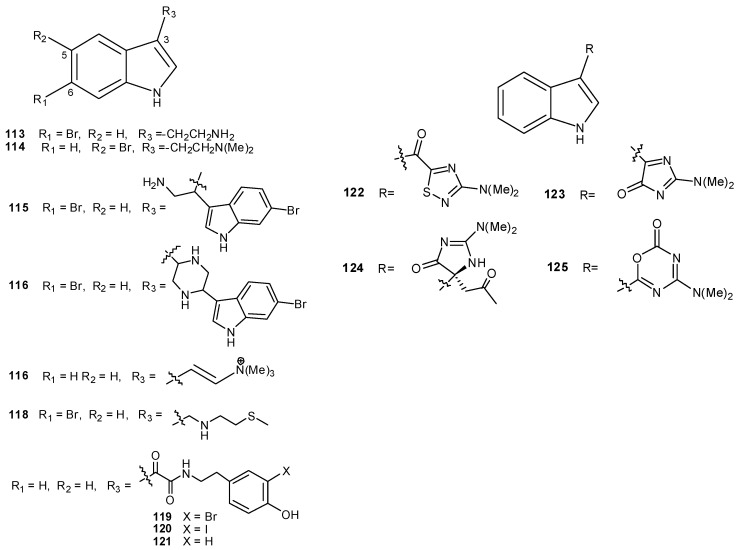
3-Substituted indoles.

A number of 3-substituted indoles have been isolated from ascidians. Examples are 6-bromotryptamine (**113**) and its derivatives **114–116**, isolated from *Didemnum candidum * [81], conicamin (**117**), from *Aplidium conicum*, with histamine-antagonistic activity [82], citorellamine (**118**), from *Polycitorella mariae*, the first indole disulfide dihydrochloride from a marine organism [83,84], and the indolyl-3-glyoxylic acid derivatives polyandrocarpamides A–C (**119–121**) [85]. *Dendrodoa grossularia *was the source of several indole alkaloids with different heterocyclic moieties linked at the 3-position (compounds **122–125**) [86,87,88,89]. Among them, alboinon (**125**) contained an oxadiazinone system, found in nature for the first time (Figure 14) [88].

**Figure 15:**
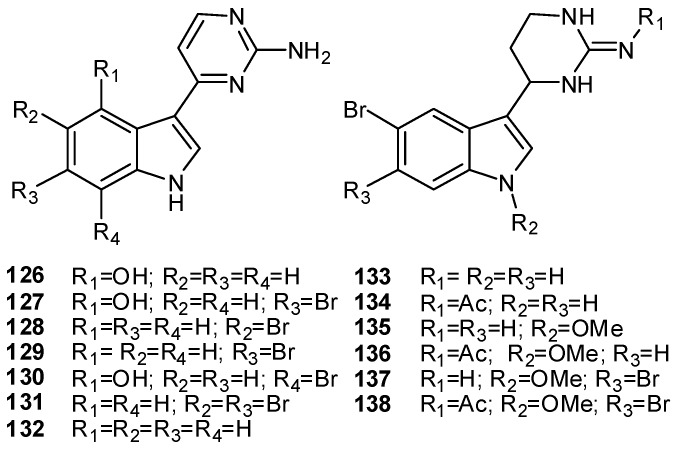
3-(2-Aminopyrimidine)-indoles (meridianins A–G, **126–132**) and their reduced forms (aplicyanins A-F, **133–138**).

Meridianins A–G (**126–132**), from *Aplidium meridianum * [90,91], are brominated and/or hydroxylated 3-(2-aminopyrimidine)-indoles differing in the bromine and /or hydroxyl substitution (Figure 15). They constitute a new family of protein kinase inhibitors, inhibiting various protein kinases such as cyclin-dependent kinases, glycogen synthase kinase-3, cyclic nucleotide-dependent kinases and casein kinase 1; they also prevent proliferation and induce apoptosis probably due to their ability to enter cells and to interfere with the activity of kinases important for cell division and death [92]. Structurally related to meridianins are aplicyanins A–F (**133–138**), from *A. cyaneum, *containing a 6-tetrahydropyrimidine substituent at C-3; these alkaloids can be considered reduced forms of the relevant meridianins and, thus, their biogenetic precursors (Figure 15) [93].

**Figure 16:**
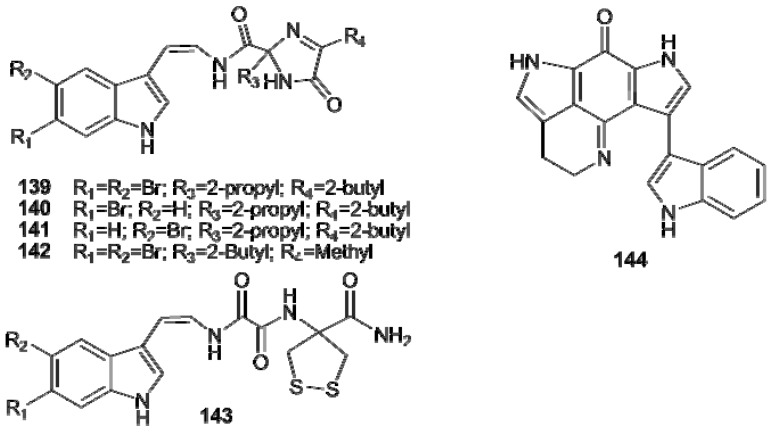
Structures of kottamides A–E (**139–143**) and wakayin (**144**).

Kottamides A–D (**139–142**), from *Pycnoclavella kottae * [94], are imidazol-4-one containing alkaloids; their biogenesis could involve stereospecific imidazolone ring formation from modified Trp-Val-Ile and Trp-Ile-Ala tripeptide precursors (Figure 16) [96]. Kottamide E (**143**) represent the first report of a natural product bearing a 4-amino-1,2-dithiolane-4-carboxylic acid residue [95]. Wakayin (**144**), from *Clavelina* sp., contains an uncommon pyrroloiminoquinone moiety; it is one of the first camptothecin (CPT)-like TOPO I inhibitors isolated from a marine organism, the indole ring linked to the bispyrroleiminoquinone core playing a significant role in this activity (Figure 16) [96].

**Figure 17:**
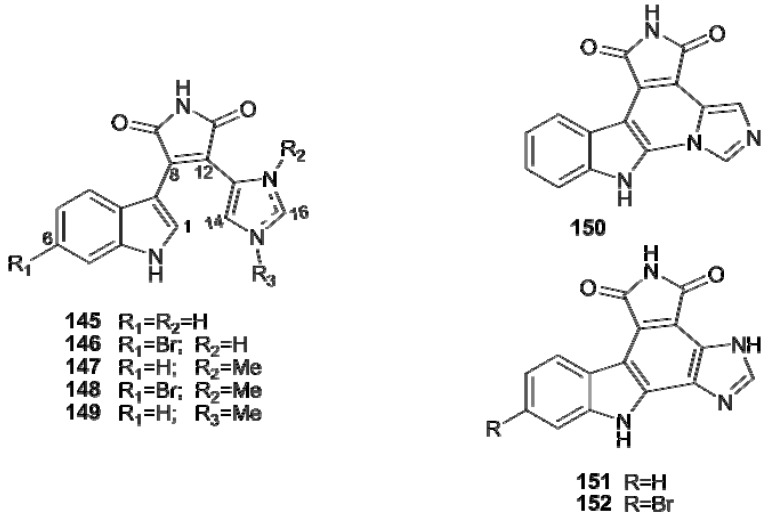
Indole-maleimide-imidazole alkaloids.

Four predator-deterrent alkaloids possessing a novel indole-maleimide-imidazole carbon skeleton, didemnimides A–D (**145–148**), were isolated from a Caribbean collection of *Didemnum conchyliatum * [97]. They were the first examples of a new alkaloid structural class and add to a relatively small group of naturally occurring maleimides. Successively, didemnimide E (**149**) was isolated from *Didemnum granulatum *collected in Brazil together with a less polar and deep purple cyclized didemnimide alkaloid, isogranulatimide (**150**) [98]. Almost at the same time, Fenical’s group independently obtained this compound from *D. conchyliatum * [99]. Compound **150** is the cyclization product of didemnimide A (**145**) formed via a C-2 indole condensation with the imidazole nitrogen and represented the first alkaloid to be isolated with this cyclized indole-maleimide-imidazole structure. Isogranulatimide (**150**) and its isomer granulatimide (**151**) could be biomimetically synthesized involving the photocyclization of **145**. Subsequently, **151** and its 6-bromo derivative **152** were obtained as naturally occurring compounds from a new collection of Brazilian *D. granulatum * [100]. Isogranulatimide (**150**) was reported as the first non-cytotoxic, specific G2 cell cycle checkpoint inhibitor, and the mechanism of this antitumor action was elucidated at molecular level (Figure 17) [98,101].

**Figure 18:**
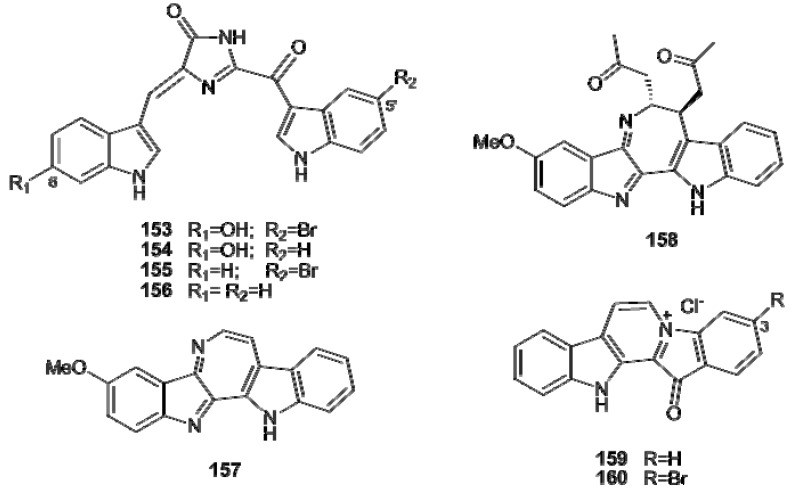
Bisindole alkaloids.

Apart from the above mentioned staurosporine derivatives **104–112**, other examples of bisindole alkaloids are rhopaladins **153–156**, from *Rhopalaea *sp., possessing an imidazolinone moiety [102], and iheyamines A (**157**) and B (**158**), from *Polycitorella *sp., with a new heteroaromatic skeleton composed of an azocine unit fused onto a bisindole system (Figure 18) [103]. The 12*H*-pyrido[1,2-a:3,4-b0]diindole ring system forms the framework of the red pigment fascaplysin (**159**), which was isolated in 1988 from the sponge *Fascaplysinopsis Bergquist* sp. and successively found in other Thorectidae sponges; this compound, together with its 3-bromoderivative (**160**), has been found in some *Didemnum* collections [62,104,105]. Fascaplysin and its derivatives exhibit a broad range of bioactivities including antibacterial, antifungal, antiviral, HIV-1-RT, p56 tyrosine kinase, antimalarial, citotoxicity against numerous cancer cell lines, specific inhibition of Cdk 4 and DNA intercalation, demonstrating a huge potential for therapeutic assays [106].

## 5. Tyrosine- and Phenylalanine-Derived Alkaloids

Tyrosine is the precursor of a wide number of alkaloids whose structures are characterized by possessing the Ar–C_2_–N subunit derived from Tyr, commonly via dopamine; often, additional Ar–C_1_ and Ar–C_2_ moieties are present, derived from a partial degradation of the amino acids Phe or Tyr. The aromatic ring of all these subunits is usually oxygenated at 4-, 3,4-, or 3,4,5-positions. The amino acid DOPA [2-amino,3-(3′,4′-dihydroxyphenyl) propionic acid], in particular, appears to play an important role in the ascidians’ metabolism, serving as the apparent precursor not only of peptide products, but also of unique alkaloid structures, such as those of lamellarins and ecteinascidins (see below).

**Figure 19:**
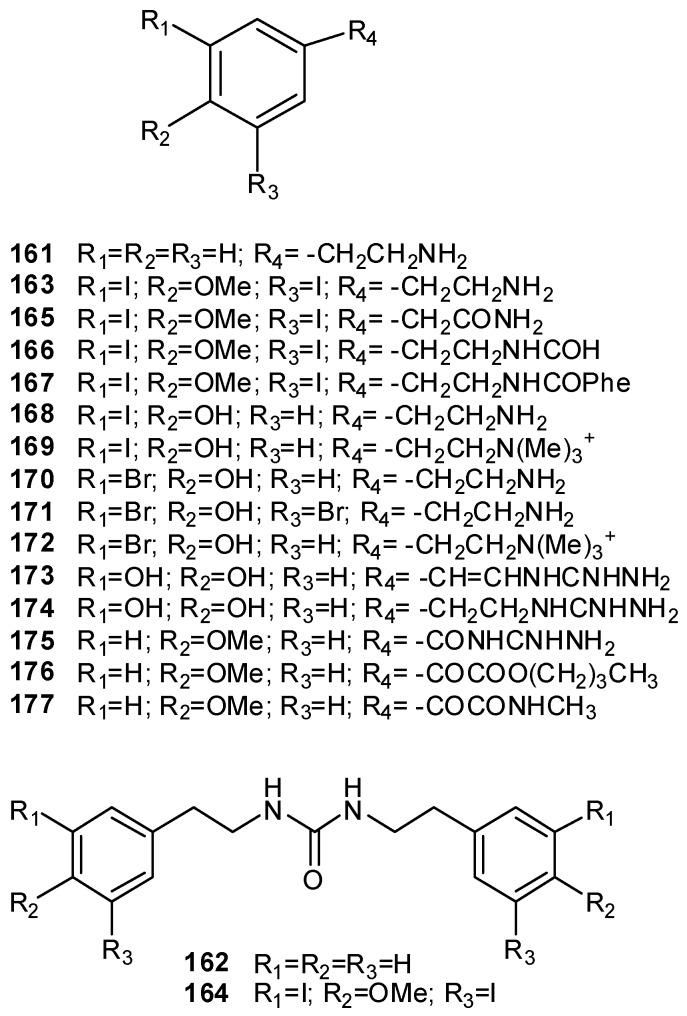
Iodinated and/or brominated tyramine derivatives.

Simple aromatic alkaloids, derived directly or indirectly from phenylalanine, tyrosine, phenylethylamine, tyramine, or dopamine, as well as complex highly condensed structures fall in the group of tyrosine- and phenylalanine-derived alkaloids. Phenylethylamine (**161**) itself and its urea derivative **162** have been found in a *Lissoclinum* sp. and in *Didemnum ternatanum*, respectively [18,107]. Iodinated and/or brominated tyramine derivatives, such as compounds **163–172**, are often found within ascidians metabolites [52,108,109,110,111,112]. The guanidine derivatives tubastrine (**173**) and its saturated analogue **174** have been found in a New Zealand collection of *Aplidium orthyum* [113]; an Australian collection of *Polycarpa aurata* contained the three *p*-methoxybenzoyl derivatives **175–177** (Figure 19) [114].

**Figure 20:**
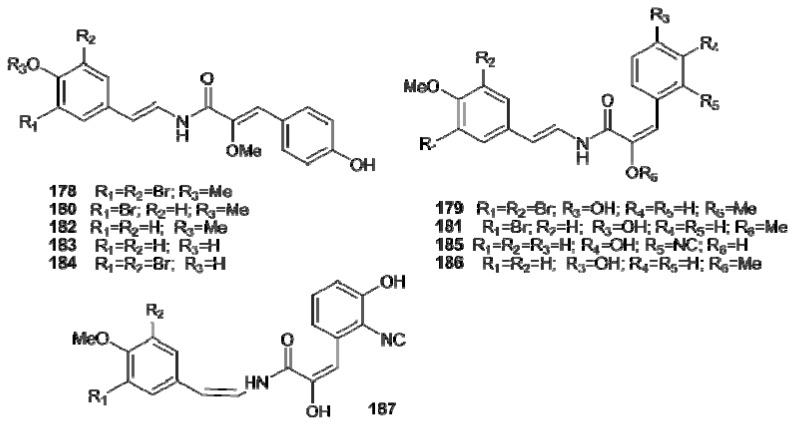
Structures of botryllamides A–J (**178–187**).

Botryllamides (A–J, **178**–**187**) are a series of brominated tyrosine derivatives isolated from several *Botryllus* species (Figure 20) [115,116,117,118]; they form a new class of selective inhibitors of ABCG2, a human ATP-binding cassette (ABC) transporter gene usually associated with multidrug resistance in cancer [117,119].

**Figure 21:**
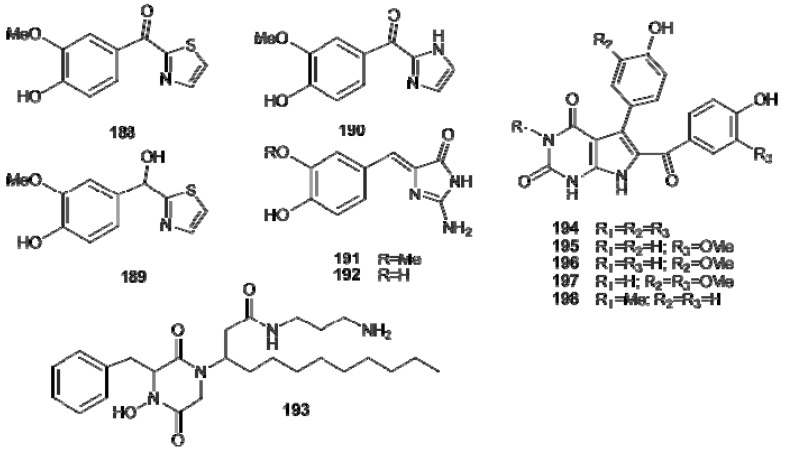
Thiazole (**188**, **189**), imidazole (**190**), 2-aminoimidazolone (**191**, **192**), 1,4diketopiperazine (**193**), and pyrrolopyrimidine (**194–198**) alkaloids.

In some alkaloids, the amino group of what initially was the amino acid residue has ended up as a part of a heteroaromatic ring. Examples are the thiazole and imidazole metabolites **188–190**, from *Aplidium pliciferum* [120] as well as the polyandrocarpamines A and B (**191** and **192**), from *Polyandrocarpa* sp. possessing a 2-aminoimidazolone moiety [121]. A condensation between phenylalanine and glycine is expected to form the 1,4-diketopiperazine unit present in etzionin (**193**), an unusual antifungal metabolite isolated from an unidentified Red Sea tunicate [122]. Rigidin (**194**), from *Eudistoma cf. rigida*, was the first pyrrolopyrimidine alkaloid to be isolated from a marine source; it exhibited calmodulin antagonistic activity for inhibition of calmodulin-activated brain phosphodiesterase [123]. Four rigidin congeners, rigidins B–E (**195–198**), have been successively isolated from *Cystodites* and *Eudistoma *species (Figure 21) [124,125].

**Figure 22:**
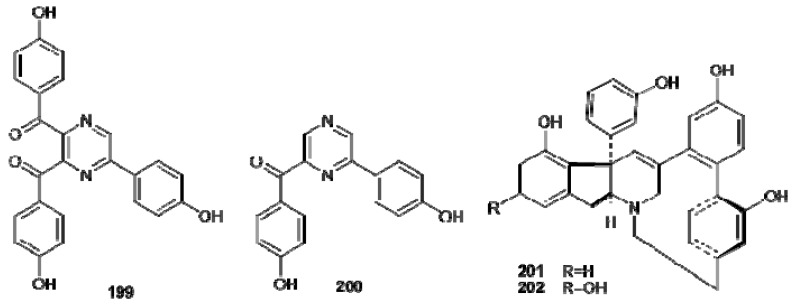
Structures of botryllazines (**199–200**) and haouamines (**201–202**).

Botryllazines A and B (**199** and **200**) have been isolated from *Botryllus leachii *(Figure 22); the structure of botryllazine A is unprecedented since three tyrosine precursors are involved in the formation of a pyrazine nucleus. It has been proposed that the biogenetic pathway leading to botryllazyne B from two tyrosine units involves amide formation and, subsequently cyclization *via* imine formation [126]. Haouamines A and B (**201** and **202**), from *Aplidium haouarianum* [127], demonstrates intriguing stereochemical features; in solution, each haouamine exists as an inseparable mixture of two interconverting isomers derived by the presence of a highly strained 3-aza-[7]-paracyclophane moiety in their structures (Figure 22). Even if one Ar–C_2_–N and three Ar–C_2_ subunits can be readily identified in the structures of haouamines, the oxygenation pattern of all these units, hydroxylated at the *meta* position with respect to the C2 chain, does not allow establishing a relationship between these alkaloids and the amino acid tyrosine. Thus, the biosynthesis of these alkaloids should imply either an unusual loss of the C4 hydroxyl group of tyrosine along the biosynthetic pathway or the involvement of an undescribed natural amino acid precursor exclusively *meta* hydroxylated [127].

The lamellarins form a group of more than 50 highly condensed DOPA- and TOPA-derived pyrrole cytotoxic alkaloids which have attracted researchers’ interest due to both their structural originality and complex mechanism of action. Lamellarins were first isolated from the prosobranch mollusk *Lamellaria *sp. in 1985 [128]. They were later extracted from and identified in various species of *Didemnum *ascidians [129,130,131,132,133,134] and sponges [135,136] collected from very diverse areas, which suggests a potential microbial link to their biosynthesis [137,138].

**Figure 23:**
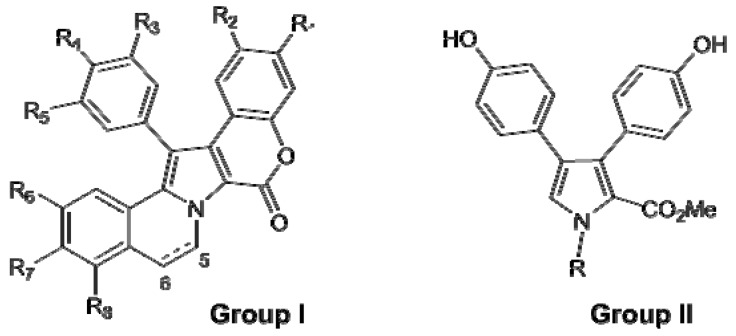
Core structures of lamellarins.

**Figure 24:**
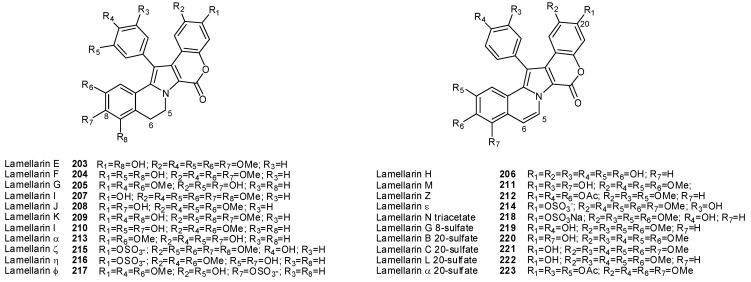
Structures of lamellarins isolated from ascidians.

The lamellarins fall into two structural groups, depending on whether the central pyrrole ring is fused (Group I) or unfused (Group II) to adjacent aromatic rings (Figure 23). Group I could be further divided into two subgroups, Ia, including compounds with an olefin at C5/C6, and Ib, with compounds in which this olefin is saturated. Each group includes derivatives in which phenolic hydroxyl groups are substituted by methoxy, sulphate or acetate functions.The lamellarins isolated from ascidians possess the hexacyclic skeleton of Group I; selected structures of ascidians’ lamellarins are reported in Figure 24.

A wide range of different biological properties are reported for this family of alkaloids, including antibiotic activity, immunomodulation, antioxidant activity [137,138,139]. A few members of the family revealed HIV-1 integrase inhibition activity, although this pharmacological activity has not been so far thoroughly explored. The HIV-1 integrase, providing the integration of the proviral DNA into host cell chromosomal DNA, constitutes an attractive target because no human cellular homologue of this enzyme exists. Lamellarin α 20-sulfate (**223**) exhibited a potent *in vitro* inhibition against the HIV-1 integrase [134]; it is able to act at two different steps of the catalytic cycle, the terminal cleavage and the strand transfer. Moreover, this compound inhibits the viral replication in cultured cells at non-toxic doses. Its sulphate group plays a critical role because the desulphated analogue lamellarin α (**213**) is inactive. However, the most common and remarkable property of the lamellarins is their capacity to inhibit the proliferation of cancer cells. The majority of lamellarins are considerably cytotoxic, with IC_50_ (or LD_50_) values in the nanomolar to micromolar range, depending on the experimental conditions and the nature of the compounds. A noticeable exception is that of the sulphated lamellarins, which are not cytotoxic presumably due to reduced cell uptake. Lamellarin D (**206**) is one of the most cytotoxic compounds of the family and, in the series of its derivatives, precise structure-activity relationships have been delineated [140]. Analogues with hydroxyl groups at both the C8 and C20 positions are the most cytotoxic, whereas the OH at C14 and methoxy groups at C13 and C21 seem to be less important to maintain the cytotoxic potential [141].

Lamellarins thus represent an important source of structures to develop anticancer drugs, although few aspects of their mechanism of actions are known, in particular their capacities to interfere with topoisomerase I and mitochondria, both contributing to their potent cytotoxicity. In particular, TOPO I has been found to be the major (but not the unique) target for lamellarin D (**206**) [142,143]; this key discovery has opened the doors to the determination of structure-function relationships and the rational design of lamellarin analogues of pharmaceutical interest. For example, the double bond between carbons 5 and 6 in the quinoline B-ring is a crucial element for topoisomerase I inhibition [142]. In addition, some lamellarins, have been reported to act as nontoxic inhibitors of acquired multidrug resistance (MDR) in various cancer cell lines. Lamellarin I (**207**) is a MDR modulator, inhibiting directly P-glycoprotein mediated drug efflux at non-lethal doses [144]. In light of their fascinating unique structures and intriguing biological properties, lamellarins still are a particularly important subject for synthetic as well as pharmacological studies [137,138,139].

**Figure 25:**
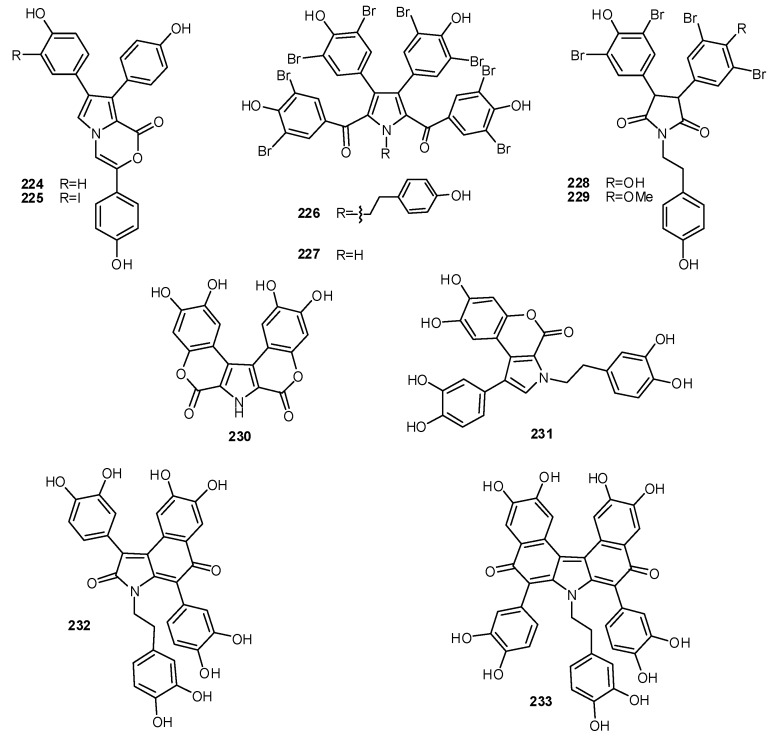
Structures of alkaloids related to lamellarins.

Pyrrole-derived alkaloids related to lamellarin include lukianols (**224–225**), isolated from an unidentified Pacific tunicate [145], polycitones (**226–227**) and polycitrins (**228–229**), from *Polycitor* species [146,147], and ningalins (**230–233**), from *Didemnum *sp. (Figure 25) [148]. The structures of the lukianols A and B contained a pyrrolooxazinone moiety; they appear to arise by the same biogenetic pathways leading to lamellarin O and P isolated from the marine sponge *Dendrilla cactos * [135]. Lukianol A (**224**) inhibited DNA synthesis in L1210 lymphocytic leukemia cell lines with less effect on RNA and protein synthesis, demonstrating a therapeutic profile very similar to current clinically used anticancer agents [145]. Ningalins A–D (**230–233**) are condensed aromatic systems with the unifying theme that all appear derived from the condensation of two, three, four, and five DOPA precursors, respectively. Although a potential role for these alkaloids in metal sequestration has not been demonstrated they are structurally related to other metal binding ascidian-derived *o*-catechols; it is thus conceivable that they too participate in the metal chelating phenomena characteristic of this class of marine invertebrates [137,145].

**Figure 26:**
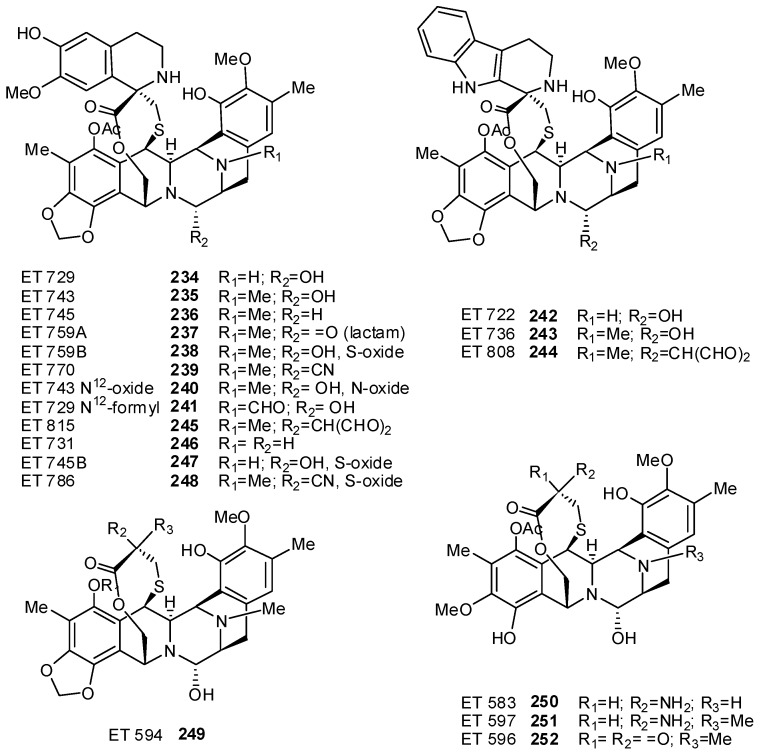
Structures of ecteinascidins.

Tetrahydroisoquinoline alkaloids are uncommon in ascidians, but the sole representatives of this class, ecteinascidins, isolated mainly from the Caribbean ascidian *Ecteinascidia turbinata*, are probably the most useful anticancer agents found to date in a marine source. The lead compound, Yondelis® (trabectedin, ET-743, **235**), is the first representative of a marine natural product to receive marketing authorization for the treatment of patients with soft tissue sarcomas (STS) [149]. Apart from the biological activity, ET-743 exhibits an extraordinary three-dimensional molecular architecture, which has made a fascinating target for the synthetic organic chemists allowing a number of original synthetic and semi-synthetic methodologies for the preparation of a wide variety of ecteinascidins to be discovered [150]. The first description and structural characterization of six new chemical entities called ecteinascidins, ET 729 (**234**), ET 743 (**235**), ET 745 (**236**), ET 759A (**237**), ET 759B (**238**), and ET 770 (**239**), was reported by the Rinehart group in 1990 of which ET-743 was the most abundant representative [151]. Simultaneously, Wright and coworkers described compounds **234** and **235** [152], but the unequivocal assignment of the absolute stereochemistry was achieved only when the X-ray crystal structures of the natural *N^12^*-oxide of ET-743 (**240**) and a synthetic O-methyl analogue of *N^12^*-formyl ET-729 (**241**) were solved [153]. Successively, a number of additional new members of this class of molecules have been isolated, such as compounds **242** and **243** [154], **244–247** [155], **248** [156], **249–252** [157] (Figure 26). The unique structure of ecteinascidins consists of a monobridged pentacyclic skeleton composed of two fused tetrahydroisoquinoline rings (subunits A and B) linked to a 10-membered lactone bridge through a benzylic sulfide linkage. Most ecteinascidins have an additional tetrahydroisoquinoline or tetrahydro-β-carboline ring (subunit C) attached to the rest of the structure through a spiro-ring. As for the biogenesis of the ecteinascidins, it has been proposed that A-B units could be formed by condensation of two DOPA-derived building blocks, and the tetrahydroisoquinoline ring in unit B is closed by condensation with a serine-(or glycine-) derived aldehyde as in the case of the related saframycins. S-Adenosylmethionine is the likely source of methyl groups at C-6, O-7, C-16, O-17, and N-12 [157]. Initially, ecteinascidins were found to be cytotoxic against L1210 leukaemia cells (IC_50_ value of 0.5 ng/mL) [151] and were later shown to possess strong *in vivo *antitumour effects in various mice models bearing P388 lymphoma, B16 melanoma, M5076 ovarian sarcoma, lewis and lX-1 human lung carcinoma, and MX-1 human mammary carcinoma xenografts [154]. ET-743 (**235**) was selected for further development; studies on its mechanism of action revealed it as the first of a new class of DNA binding agents with a complex, transcription-targeted mechanism of action. Trabectedin binds to the minor groove of DNA with preference for GC-rich triplets and subsequently forms covalent adducts with the N2-position through its carbinolamine moiety [158,159,160]. ET-743, under the trade name Yondelis, was approved for the treatment of refractory soft-tissue sarcomas by the European Commission in July 2007; the currently ongoing Phase III trials along with the already existing clinical evidence may provide enough data for the Food and Drug Administration for an approval in the US [161]. Based on the preclinical results, trabectedin is also being developed for ovarian, prostate, lung, breast and pediatric cancers [149,162,163].

## 6. Lysine-Derived Alkaloids

Alkaloids based on a 2-amino-3-hydroxyoctadecane moiety, generally referred to as lysine-derived metabolites, have been found in several genera of ascidians. Monocyclic examples are the piperidine alkaloids, which are among the most abundant metabolites of terrestrial plants, but there are relatively few examples isolated from marine organisms.

**Figure 27:**
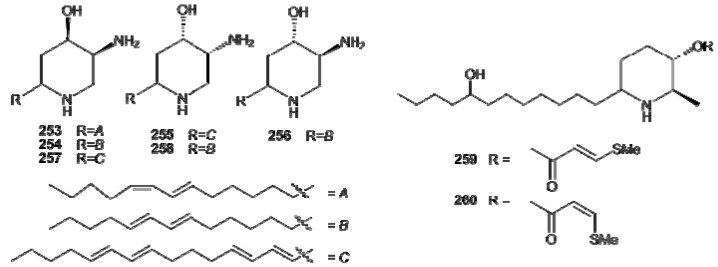
Structures of piperidine alkaloids pseudodistomins (**253–258**) and uoamines (**259**, **260**).

Pseudodistomins A (**253**) and B (**254**), from *Pseudodistoma kanoko*, represent the first piperidine alkaloids obtained from marine sources [164]. The structure of the side chains of **253** and **254** has been revised after the first disclosure of the molecules [165,166], while the stereostructure of the piperidine nucleus has been established by synthesis of their tetrahydroacetyl derivatives [167,168]. Other members of the group have been successively isolated, from other *Pseudodistoma* species, including pseudodistomin C (**255**), with the absolute configuration at C-4 and C-5 opposite to those found in **253** and **254** [169], and pseudodistomins D–F (**256–258**) (Figure 27) [170]. Pseudodistomins A and B exhibited cytotoxic activity with calmodulin antagonistic activity; pseudodistomins B–F were found to be active in a cell-based assay for DNA damage induction. Uoamines A (**259**) and B (**260**) also are monocyclic piperidine alkaloids isolated form *Aplidium uouo*; in these compounds, the 3-hydroxyl group present on the piperidine nucleus is esterified by (*E*)- or (*Z*)-3-thiomethylacrylic acid, respectively (Figure 27) [171].

**Figure 28:**
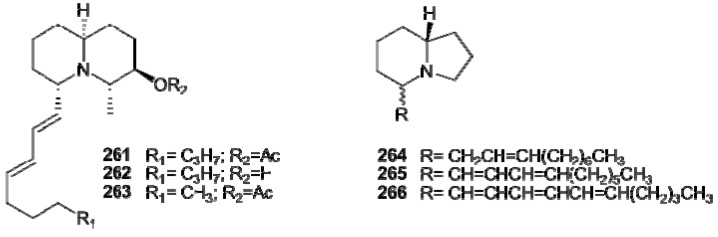
Structures of quinolizidine (**261–263**) and indolizidine (**264–266**) alkaloids.

Bi-and tricyclic members of the lysine-derived alkaloids group have been most often found in *Clavelina *species and include quinolizidines and indolizidines derivatives. Clavepictines A (**261**) and B (**262**) and pictamine (**263**) are homologous quinolizidine alkaloids isolated from Bermudan and Venezuelan collections of the tunicate *C. picta**, *** respectively [172,173]. With the use of NOE experiments, **261** was determined to have a *cis* ring junction in the quinolizidine moiety, with the decadienyl side chain in the equatorial orientation, and the methyl and acetoxy substituents oriented *trans**-***diaxial. An X-ray analysis of clavepictine B (**262**) confirmed the proposed relative stereochemistry and conformation. Both clavepictines inhibited the growth of murine leukemia and human solid tumor cell lines at concentrations less than 9 μg/mL. Pictamine (**263**) is a bis-nor analogue of clavapictine A, bearing two less carbons on the side-chain. *C. picta* has also been the source of a series of indolizidine metabo1ites, piclavines A–C (**264–266**) (Figure 28) [174]. They consist of three series of isomeric compounds**, **differing in the number of double bonds on the side chain. Each group is composed of an inseparable mixture of stereoisomers, which differ at the C2 chiral center, as well as at each of the double bonds. The piclavines are the first indolizidines isolated from a marine source; they exhibited antifungal and antimicrobial activity against Gram-positive bacteria.

**Figure 29:**
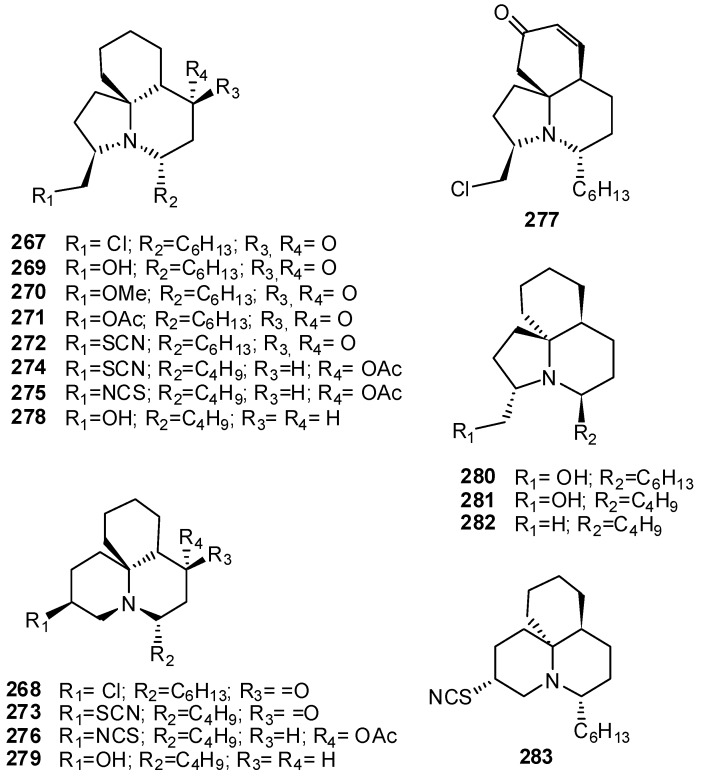
Structures of tricyclic lysine-derived alkaloids from ascidians.

Tasmanian collections of *C. cylindrica *yielded the tricyclic alkaloids cylindricines A–K (**267–277**) [175,176,177]; cylindricine B (**268**) represented the first example of the new perhydropyrido[2,1-*j*]quinoline ring system, while cylindricine A (**269**) is the first perhydropyrrolo-[2,1-*j*]quinoline known from nature (Figure 29). The skeleton of cylindricines A (**267**), C–I (**269–275**), and K (**277**) is closely related to the indolizidine system of piclavines by having an extra 6-membered carbocyclic fused ring. In the same way, structures of cylindricines B (**268**) and J (**276**) are related to the quinolizidine system of the clavepictines and pictamines. Cylindricines F (**272**) and G (**273**) are the first thiocyanates from an ascidian. Closely related to cylindricines A and B, are polycitorols A (**278**) and B (**279**), isolated from a marine ascidian of the family Polycitoridae [178]; they lack C-4 oxygenation found in cylindricines and have a OH group instead of a chlorine atom at C-13 and a butyl instead of a hexyl appendage at C-2 (Figure 29). Other analogous metabolites are lepadiformines A–C (**280–282**) isolated from *C. lepadiformis* and *C. moluccensis* [179,180] and fasicularin (**283**) from *Nephteis fasicularis * [181]. The initially reported structure of lepadiformine A was revised through its total synthesis; in the same study fasicularin (**283**) was also synthesized, allowing its absolute configuration to be assigned (Figure 29) [182]. Early biological experiments on fasicularin suggested that its cytotoxic properties may stem from its ability to damage cellular DNA [181]. Successive studies revealed fasicularin as the first natural product found to generate a DNA-alkylating aziridinium ion via a mechanism analogous to the clinically used anticancer drugs mechlorethamine, melphalan, and chlorambucil [183]. Biological studies on lepadiformines showed that they have marked effects on the cardiovascular system when tested on frog atrial myocytes, the potency varying with the alkaloid structure [180].

## 7. Protoalkaloids

**Figure 30:**
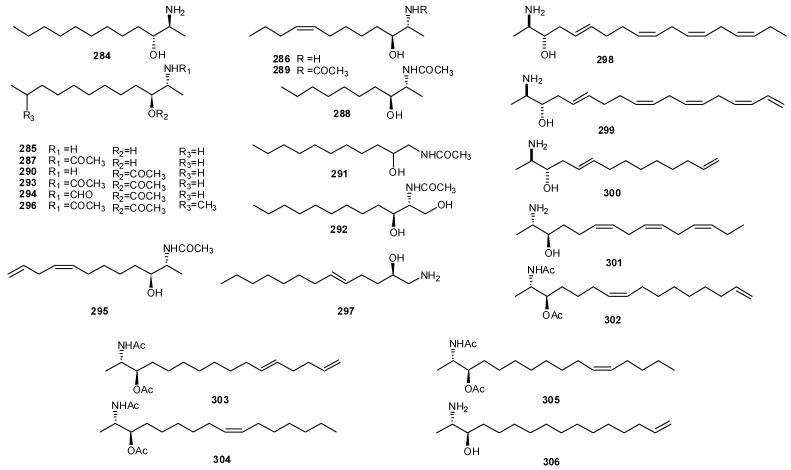
Structures of linear2-aminoalkanols.

Ascidians have been also the source of protoalkaloids, simple amines in which the nitrogen is not in a heterocyclic ring. *Clavelina* and *Pseudodistoma* genera have been prolific in the production of linear 2-aminoalkanols and their unsaturated and/or acetylated derivatives. Structurally, these compounds are related to the sphingosine derivatives, which are central structural elements of sphingolipids and important constituents of the lipid portion of cell membranes in living organisms. The carbon chain length of these sphingolipid derivatives vary from C12 to C18 amino alcohols. Examples are the C12 saturated amino alcohol (2*S*,3*R*)-2-aminododecanol-3-ol (**284**) isolated from *C. oblonga* [184], the clavaminols A–N (**285–296**), twelve saturated and unsaturated cytotoxic sphingoids isolated from the Mediterranean *C. phlegraea* [185,186], and the antifungal 2-amino alcohol **297** isolated from an Australian *Didemnum* sp. (Figure 30) [187]. Crucigasterins 277, 275 and 225 (**298–300**) and A–E, and obscuraminols A–F (**301–306**) are examples of polyunsaturated 2-amino-3-alkanols; they have been isolated as their diacetyl derivative from *P. crugigaster * and *P. obscurum*, respectively (Figure 30) [188,189].

**Figure 31:**
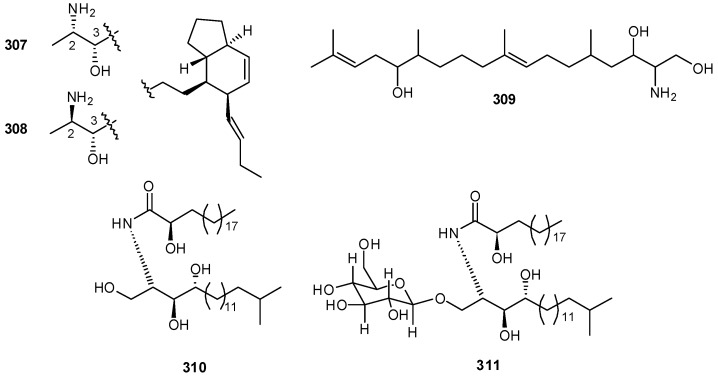
Structures of amaminols (**307**, **308**), aplidiasphingosine (**309**), and lipids **310** and **311**.

Two bicyclic amino alcohols, amaminols A and B (**307–308**) have been isolated from an unidentified tunicate of the family Polyclinidae [190]. Total synthesis of amaminol A (**307**) allowed to establish its absolute stereochemistry [191]. Other sphingosine-related compounds are aplidiasphingosine (**309**), and the two lipids (**310–311**) isolated from *Cystodytes* cf. *dellechiajei *as PLA2 inibitors (Figure 31) [192,193].

**Figure 32:**
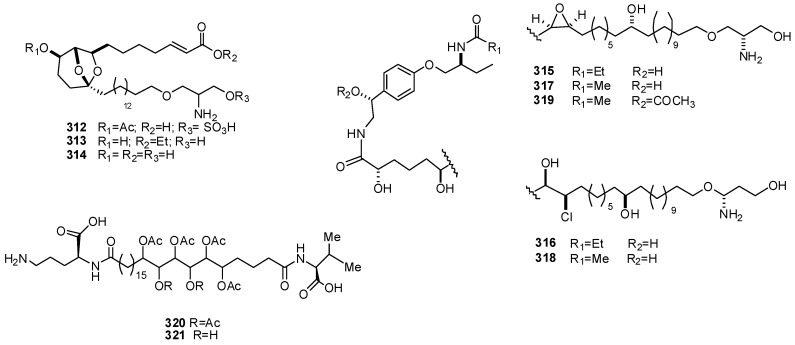
Structures of didemniserinolipids (**312–314**), shishididemniols (**315–319**), and sagittamides (**320–321**).

Didemniserinolipids A–C (**312–314**), from a tunicate of the family Didemnidae [194], are unprecedented antibacterial serinolipids, compounds containing a unique serinol component and a 6,8-dioxabicyclo[3.2.1]octane core. After its first disclosure, structure of (+)-didemniserinolipid B (**313**) was revised, as that shown, through its synthesis, which allowed also to assign to the compound the 8*R*,9*R*,10*R*,13*S*,31*S* absolute stereochemistry (Figure 32) [195]. Further serinolipid derivatives, shishididemniols A–E (**315–319**) have been isolated as antibacterial constituents of a tunicate of the family Didemnidae [196,197]; they are complex lipids with tyramine-derived tether and two serinol units (Figure 32). An unidentified tunicate from Pohnpei Micronesia yielded sagittamides A (**320**) and B (**321**), C26 dicarboxylic acids that acylate terminal L-valine and L-ornithine groups [198] and contain an unprecedented internal *O*-hexacetyl-1,2,3,4,5,6-hexaol moiety (Figure 32). The stereochemistry of sagittamide A has been extensively investigated; its absolute configuration has been established through a detailed ^1^H NMR analysis of the two remote diastereomers, followed by doping experiments of them with the authentic natural product [199].

## 8. Dimeric Steroidal Alkaloids

The ritterazines (A–Z, **322–347**), isolated from *Ritterella tokioka*, form a family of dimeric steroidal alkaloids [200,201,202,203,204]. They are closely related in structure to cephalostatins, isolated from the marine worm *Cephalodiscus gilchristi * [205]; cephalostatin 1 has been proved to be one of the most powerful cancer cell growth inhibitors with an ED50 value of 0.1–0.001 pM.

**Figure 33:**
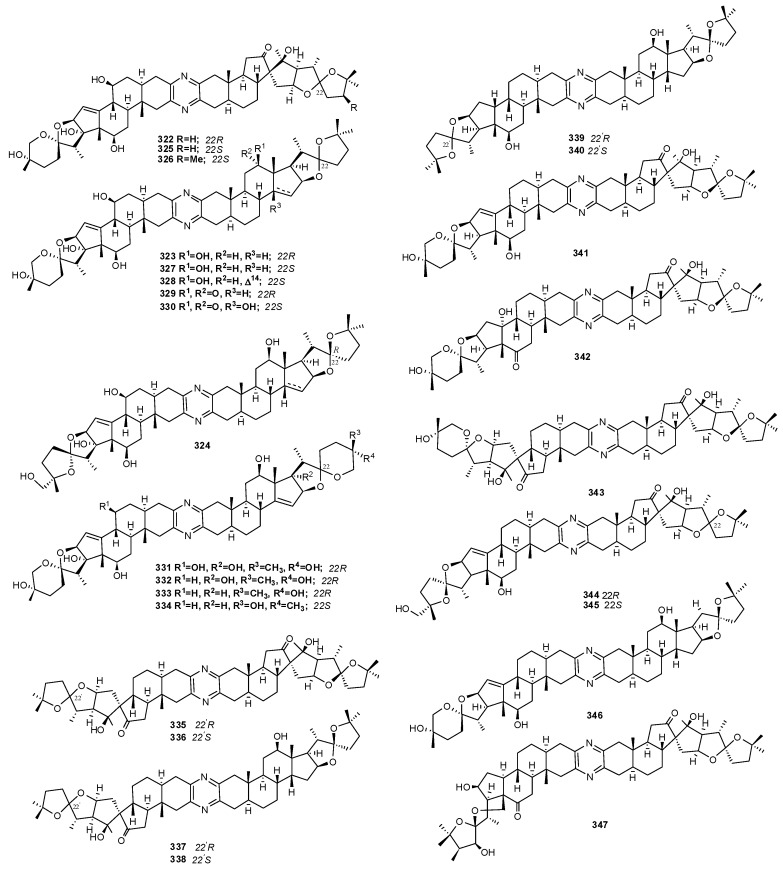
Structures of ritterazines A–Z (**323–347**).

The ritterazines and cephalostatines share many common structural features. Both consist of two highly oxygenated C27 steroid units fused via a pyrazine ring at C-2 and C-3; both chains of the steroid units usually form either 5/5 or 5/6 spiroketals. While cephalostatins in general are more oxygenated on the right side, the ritterazines have the more oxygenated left side. Hydroxyl groups are seen at C-12, C-17, C-23, C-26, C-12′, and C-23′ in the cephalostatins, whereas C-12, C-7′, C-12′, C-17′, and C-25′ are hydroxylated in the ritterazines (Figure 33) [206].

Although less potent than cephalostatins, ritterazines also possess high inhibitory activity against a series of human cancer cell lines. Not surprisingly, the most active constituent of *R. tokioka *(ritterazine B, **323**) contains nearly the same right-hand side steroidal unit (**323**: no 17*R*-hydroxy moiety) as the most active cephalostatins. A COMPARE pattern-recognition analysis gave correlation coefficients of ~0.9 between cephalostatins and ritterazines in NCI-10 cell lines, suggesting they share the same mechanism. However, the antineoplastic mechanism of the cephalostatins is presently largely unknown. The fingerprint of cephalostatin activity in the NCI 60-tumor panel is quite different from other known anticancer agents, likely indicating a new mechanism of action. Recent studies revealed that cephalostatin 1 affects cells by disrupting the mitochondrial transmembrane potential and, thus, inducing apoptosis [207]. Pro-apoptotic properties have also been demonstrated for ritterazine B (**323**) although apoptosis induced by this compound appeared to be independent of the caspase pathway. Neither cleavage nor degradation of caspase targets was indeed observed, indicating that ritterazine B might be a potent inducer of apoptosis acting via a novel antimitotic mechanism [208]. The relative simplicity of ritterazines promises greater synthetic accessibility with probable retention of significant bioactivity. Isolation of closely related cephalostatins and ritterazines from different phyla raises questions as to the true origin of bissteroidal pyrazines; a shared symbiontic microorganism could be responsible for the biosynthesis of these compounds [207].

## 9. Conclusions

An intensive research effort during the last 25 years has generated an impressive number of alkaloids isolated from marine ascidians, which remain unique among marine invertebrates in that they overwhelmingly produce this kind of metabolites. Many of these compounds exhibited biomedically important activities; among them, cytotoxicity is the most frequently listed activity. Unfortunately, the chemical ecology of these organisms has not received significant attention and several intriguing aspects of their chemistry still remain unsettled. These include whether symbionts, which are commonly associated with ascidians, play a role in their secondary metabolism, and what purpose these compounds serve for the producing organism in nature.
